# *Tremella fuciformis* polysaccharides induce ferroptosis in Epstein-Barr virus-associated gastric cancer by inactivating NRF2/HO-1 signaling

**DOI:** 10.18632/aging.205457

**Published:** 2024-01-19

**Authors:** Wencheng Kong, Xinchun Liu, Hangzhang Zhu, Sixing Zheng, Guang Yin, Panpan Yu, Yuqiang Shan, Shenglin Ma, Rongchao Ying, Huicheng Jin

**Affiliations:** 1 Department of Gastroenterological Surgery, Affiliated Hangzhou First People’s Hospital, Zhejiang University School of Medicine, Hangzhou 310006, Zhejiang Province, P.R. China; 2 Zhejiang Province Key Laboratory of Anticancer Drug Research, Institute of Pharmacology and Toxicology, College of Pharmaceutical Sciences, Zhejiang University, Hangzhou 310058, Zhejiang Province, P.R. China; 3 Translational Medicine Research Center, Key Laboratory of Clinical Cancer Pharmacology and Toxicology Research of Zhejiang Province, Affiliated Hangzhou First People’s Hospital, Zhejiang University School of Medicine, Hangzhou 310006, Zhejiang Province, P.R. China

**Keywords:** *Tremella fuciformis* polysaccharides, ferroptosis, Epstein-Barr virus-associated gastric cancer, NRF2/HO-1

## Abstract

Approximately 10% of gastric cancers are associated with Epstein–Barr virus (EBV). *Tremella fuciformis* polysaccharides (TFPs) are characterized by antioxidative and anti-inflammatory effects in different diseases. However, whether TFP improves EBV-associated gastric cancer (EBVaGC) has never been explored. The effects of TFP on EBV-infected GC cell viability were determined using a CCK-8 assay and flow cytometry. Western blotting and RT–qPCR were performed to explore the expression of ferroptosis-related proteins. The CCK-8 assay showed that TFP decreased EBV-infected GC cell viability in a dose- and time-dependent manner. Flow cytometry assays indicated that TFP significantly induced EBV-infected GC cell death. TFP also reduced the migratory capacity of EBV-infected GC cells. Furthermore, treatment with TFP significantly increased the mRNA levels of PTGS2 and Chac1 in EBV-infected GC cells. Western blot assays indicated that TFP suppressed the expression of NRF2, HO-1, GPX4 and xCT in EBV-infected GC cells. More importantly, overexpression of NRF2 could obviously rescue TFP-induced downregulation of GPX4 and xCT in EBV-infected GC cells. In summary, we showed novel data that TFP induced ferroptosis in EBV-infected GC cells by inhibiting NRF2/HO-1 signaling. The current findings may shed light on the potential clinical application of TFP in the treatment of EBVaGC.

## INTRODUCTION

Gastric cancer (GC) is the fifth most common cancer and the third leading cause of death worldwide [1]. Although treatment strategies for GC have been significantly improved, the overall survival rate is still poor due to its high rate of recurrence, metastasis and poor response to chemotherapy and radiotherapy [2, 3]. Epstein–Barr virus (EBV) was the first tumorigenic DNA virus to be identified, and approximately 90% of the population has latent EBV infection, which is also associated with gastric cancer in addition to the development of Hodgkin's lymphoma and nasopharyngeal carcinoma [4]. Studies have found that EBV-associated gastric cancer (EBVaGC) accounts for approximately 10% of gastric cancers worldwide and that EBV infection is positively associated with the risk of gastric cancer [4, 5]. More than 95% of patients with gastric cancer are found to have a typical history of *Helicobacter pylori* (*H. pylori*) infection, and EBV infection can promote the development of gastric cancer caused by H. pylori infection [6]. A recent study found that when normal gastric epithelial cells are infected with EBV, the intracellular microenvironment undergoes significant changes thereby promoting GC [7]. EBV has been reported to interact with H. pylori to increase gankyrin as well as downstream alterations in cancer gene expression, which in turn promotes malignant proliferation and migration of gastric cancer cells [8]. Therefore, to improve the prognosis of patients with EBVaGC, researchers need to further study the underlying molecular mechanisms and potential therapeutic methods.

Ferroptosis is a newly identified form of cell death that is induced via iron overload and elevation of lipid reactive oxygen species (ROS) [9–11]. In contrast to apoptosis, autophagy, and necrosis, ferroptosis can be induced by specific inducers, including eradicators of Ras and ST (erastin) and Ras selective lethal 3 (RSL3) [12]. Morphologically, cells undergoing ferroptosis have intact cytosolic membranes, normal-sized nuclei, no chromatin condensation, dense mitochondria, and residual cristae. Biochemically, cells undergoing ferroptosis exhibit elevated Fe^2+^ levels, increased ROS levels, inactivation or depletion of glutathione peroxidase 4 (GPX4), inhibition of cystine/glutamate transporter protein SLC7A11 (also xCT, system xc-), and accumulation of lipid metabolites [6]. The transcription factor nuclear factor erythroid 2-related factor 2 (NRF2) is a key member of endogenous antioxidant defense systems [13, 14]. Upregulation of NRF2 expression inhibits ferroptosis, and reduction of NRF2 promotes ferroptosis [15]. It has been demonstrated that NRF2 promotes the transcription of xCT and GPX4, which are important negative regulators of ferroptosis [16–18]. Other downstream antioxidant enzyme-encoding genes, such as HO-1, are also regulated by NRF2 [18]. Appropriate doses of HO-1 have been shown to enable bilirubin to scavenge oxygen radicals to resist lipid peroxidation [19]. Therefore, NRF2-HO-1/GPX4/xCT-associated ferroptosis is an important tumor suppressor mechanism and may be a potential therapeutic approach for EBVaGC.

*Tremella fuciformis* polysaccharides (TFP) are the active ingredients of *Tremella fuciformis*, which is a traditionally edible mushroom [20]. Increasing evidence has indicated important antioxidative and anti-inflammatory effects of TFP in different diseases [20, 21]. For instance, TFP suppresses LPS-induced oxidative stress and inflammation by inactivating NFκB signaling in macrophages [20]. TFP also protects against dextran sulfate sodium (DSS)-induced colitis in mice by inducing an anti-inflammatory response [21]. TFP has been found to improve a variety of digestive disorders [22, 23]. In methionine-choline-deficient non-alcoholic disease NAFLD mice, TFP alleviates NAFLD by attenuating hepatic lipid accumulation and reducing expression of inflammation-related genes [22]. TFP also promotes repair of the intestinal and mucus barriers ameliorating ulcerative colitis [23]. However, whether TFP improves GC or EBVaGC has not been reported.

In the present study, we first explored the role of TFP in the progression of EBVaGC in *in vitro* EBV-infected GC cells. By elucidating the potential mechanism, we sought to gain novel insights into whether and how TFP suppresses EBV-infected GC cells.

## RESULTS

### EBV-infected AGS and MKN45 cells are sensitive to erastin-induced ferroptosis

**Figure 1:**
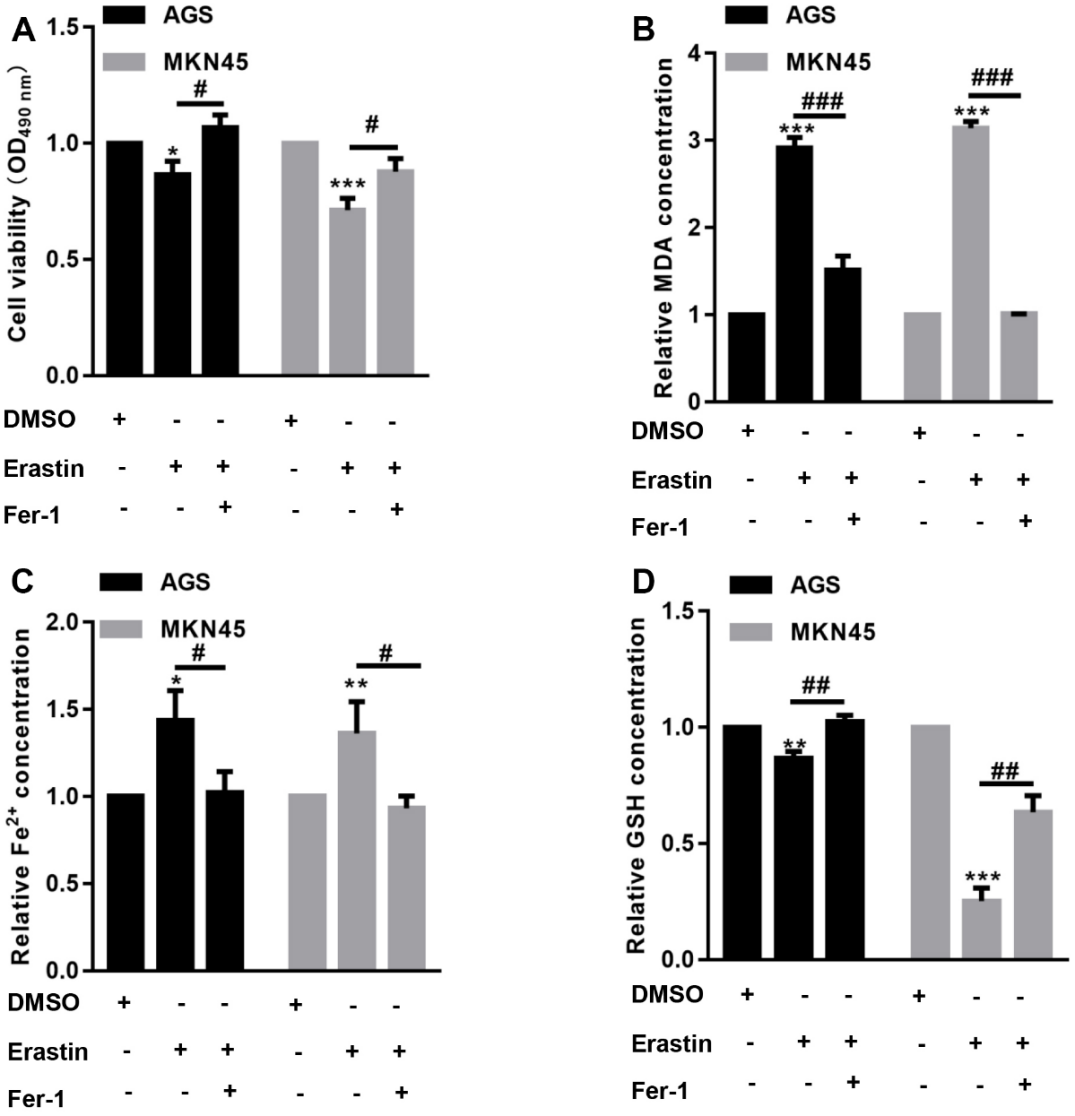
**EBV-infected AGS and MKN45 cells are sensitive to erastin-induced ferroptosis.** EBV-infected AGS and MKN45 cells were preincubated with 1 μM Fer-1 for 1 h and then treated with or without 10 μM erastin for 24 h. (**A**) CCK-8 assay demonstrated that erastin reduced EBV-infected AGS and MKN45 cell viability, but Fer-1 partially abolished these effects. The erastin-induced upregulation of MDA (**B**) and Fe^2+^ (**C**) levels was reversed by Fer-1 pretreatment in both EBV-infected AGS and MKN45 cells. (**D**) Erastin decreased intracellular GSH levels, but Fer-1 elevated GSH levels in EBV-infected AGS and MKN45 cells. ^*^p<0.05, ^**^p<0.01, ^***^p<0.001 vs. DMSO; ^#^p<0.05, ^##^p<0.01, ^###^p<0.001 vs. erastin.

The CCK-8 assay showed that erastin significantly decreased EBV-infected AGS and MKN45 cell viability, but preincubation with Fer-1 reversed the erastin-induced reduction in EBV-infected AGS and MKN45 cell viability (Figure 1A). Furthermore, we found that the levels of MDA and Fe^2+^ were increased after erastin treatment in both EBV-infected AGS and MKN45 cells, while erastin decreased GSH contents in both EBV-infected AGS and MKN45 cells (Figure 1B–1D). In comparison, preincubation with Fer-1 obviously abolished the effects induced by erastin in both EBV-infected AGS and MKN45 cells (Figure 1B–1D). These observations indicated that EBV-infected AGS and MKN45 cells were sensitive to erastin-induced ferroptosis.

### TFP decreased EBV-infected AGS and MKN45 cell viability in a dose- and time-dependent manner

**Figure 2:**
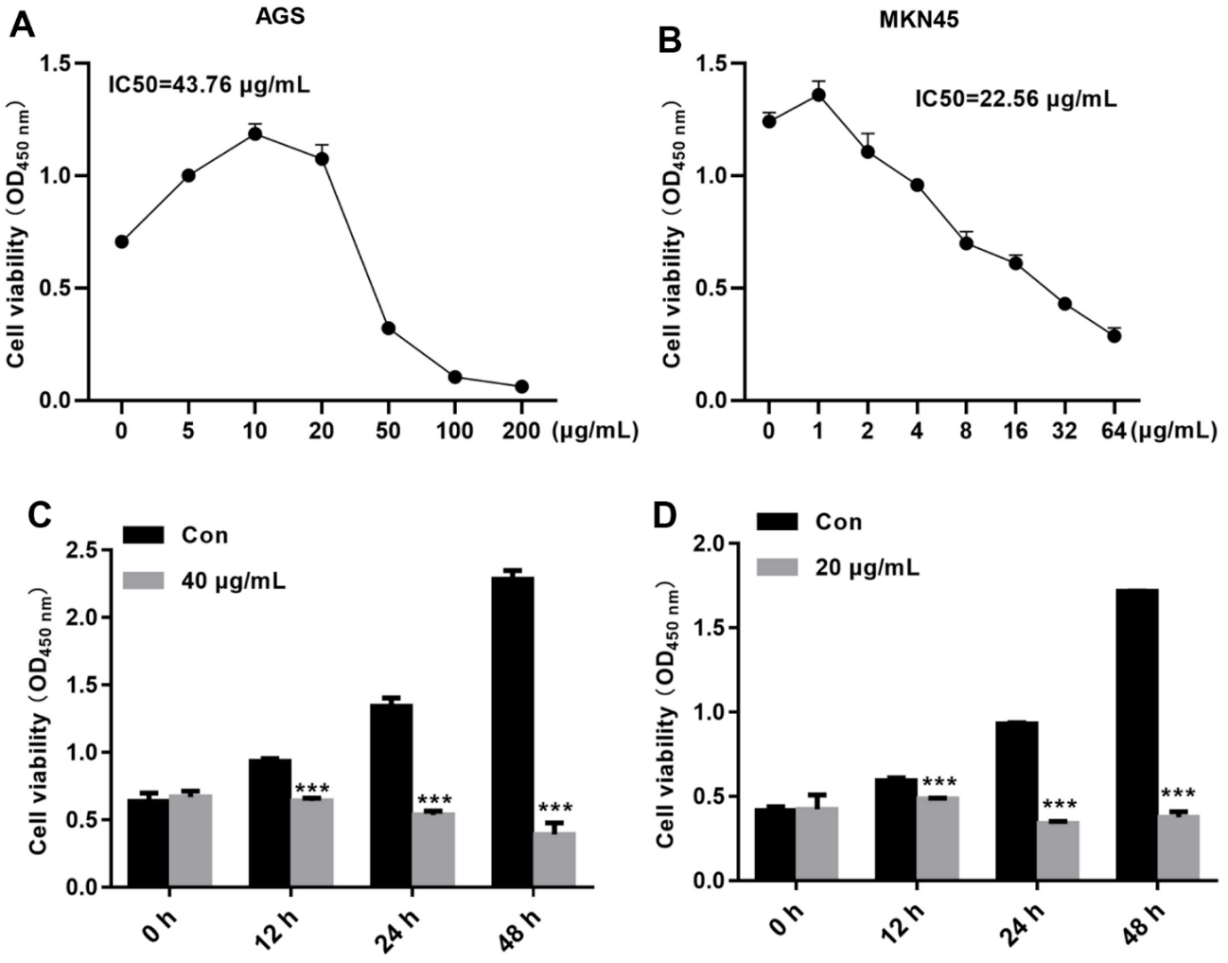
**TFP decreased EBV-infected AGS and MKN45 cell viability in a dose- and time-dependent manner.** EBV-infected AGS and MKN45 cells were treated with 0, 5, 10, 25, 50, 100, or 200 μg/mL TFP or 0, 1, 2, 4, 6, 8, 16, 32, or 64 μg/mL TFP for 24 h. CCK-8 assays showed that TFP significantly decreased AGS (**A**) and MKN45 (**B**) cell viability. EBV-infected AGS and MKN45 cells were treated with 40 μg/mL and 20 μg/mL TFP for 12 h, 24 h, 48 h and 72 h. CCK-8 assays demonstrated that TFP decreased AGS (**C**) and MKN45 (**D**) cell viability in a time-dependent manner. ***p<0.001 vs. con.

To determine whether TFP inhibits the growth of gastric cancer cells, a CCK-8 assay was carried out. Our data demonstrated that TFP exhibited strong growth inhibitory effects on EBV-infected AGS and MKN45 cells (Figure 2A, 2B). The IC50 values for EBV-infected AGS and MKN45 cells were 43.76 μg/mL and 22.56 μg/mL, respectively (Figure 2A, 2B). We then explored the effects of 40 μg/mL and 20 μg/mL TFP on EBV-infected AGS and MKN45 cells at different time points. The results showed that 40 μg/mL and 20 μg/mL TFP suppressed EBV-infected AGS and MKN45 cell viability at 12 h, 24 h, 48 h and 72 h (Figure 2C, 2D). Collectively, TFP inhibited the growth of both EBV-infected AGS and MKN45 cells in a dose- and time-dependent manner.

### TFP induced cell death and suppressed cell migration in EBV-infected AGS and MKN45 cells

**Figure 3:**
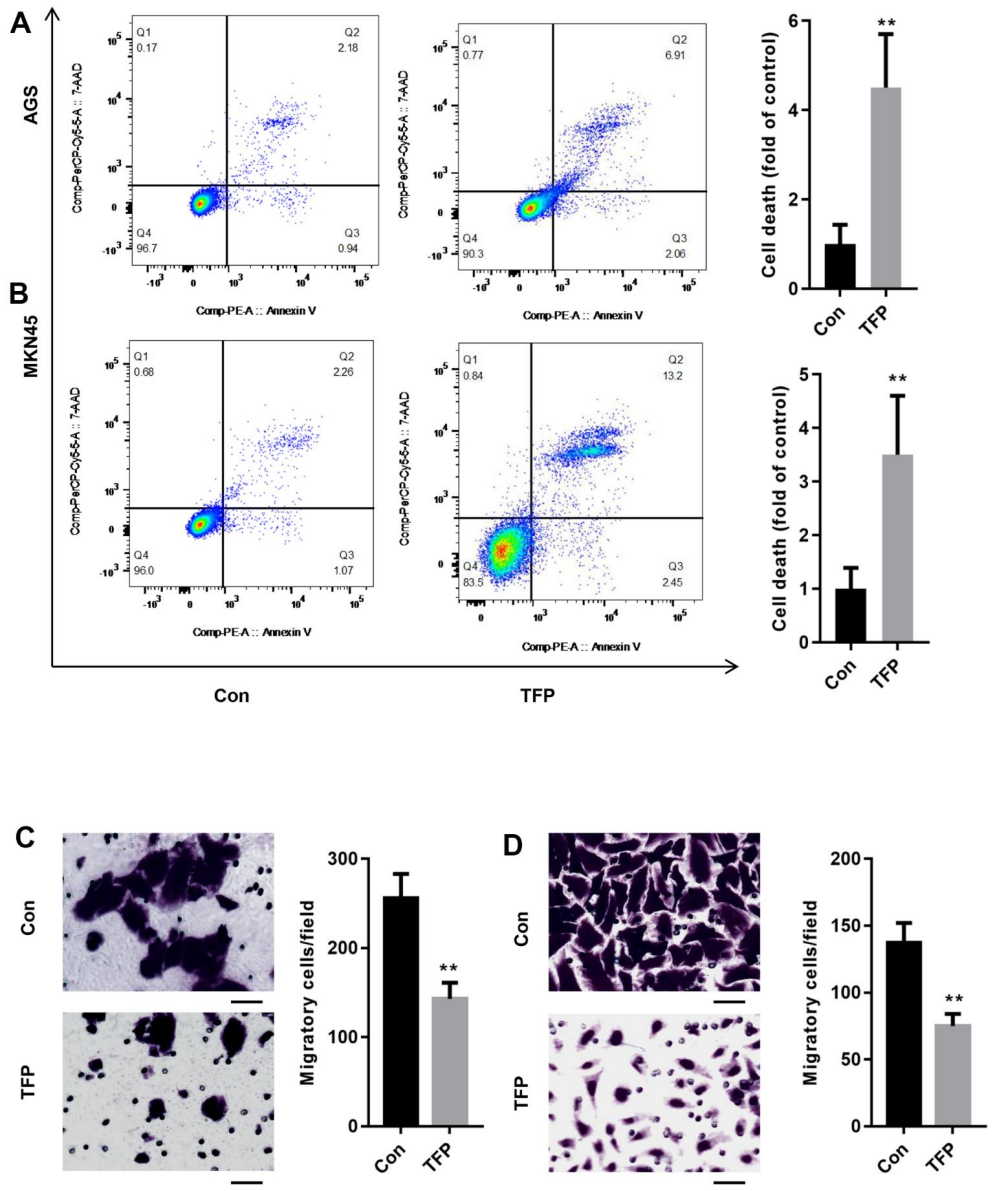
**TFP induced cell death and suppressed cell migration in EBV-infected AGS and MKN45 cells.** EBV-infected AGS and MKN45 cells were treated with 40 μg/mL and 20 μg/mL TFP for 24 h. Flow cytometry showed that TFP significantly elevated cell death in EBV-transfected AGS (**A**) and MKN45 (**B**) cells. Transwell assays demonstrated that TFP suppressed EBV-transfected AGS (**C**) and MKN45 (**D**) cell migration. **p<0.01, ***p<0.001 vs. con.

After incubation with TFP for 24 h, the effects of TFP on cell death were explored using flow cytometry. As shown in Figure 3A, 3B, TFP significantly enhanced the number of dead AGS (24.5±3.8 vs. 8.7±2.1) and MKN45 (3.9±2.8 vs. 36.5±5.6) cells compared with the number of dead control cells. Meanwhile, TFP induced a remarkable reduction in the number of migratory AGS (143±18 vs. 256±27) and MKN45 (75±9 vs. 137±15) cells relative to the vehicle-treated controls (Figure 3C, 3D).

### TFP induced ferroptosis in EBV-infected AGS and MKN45 cells

**Figure 4:**
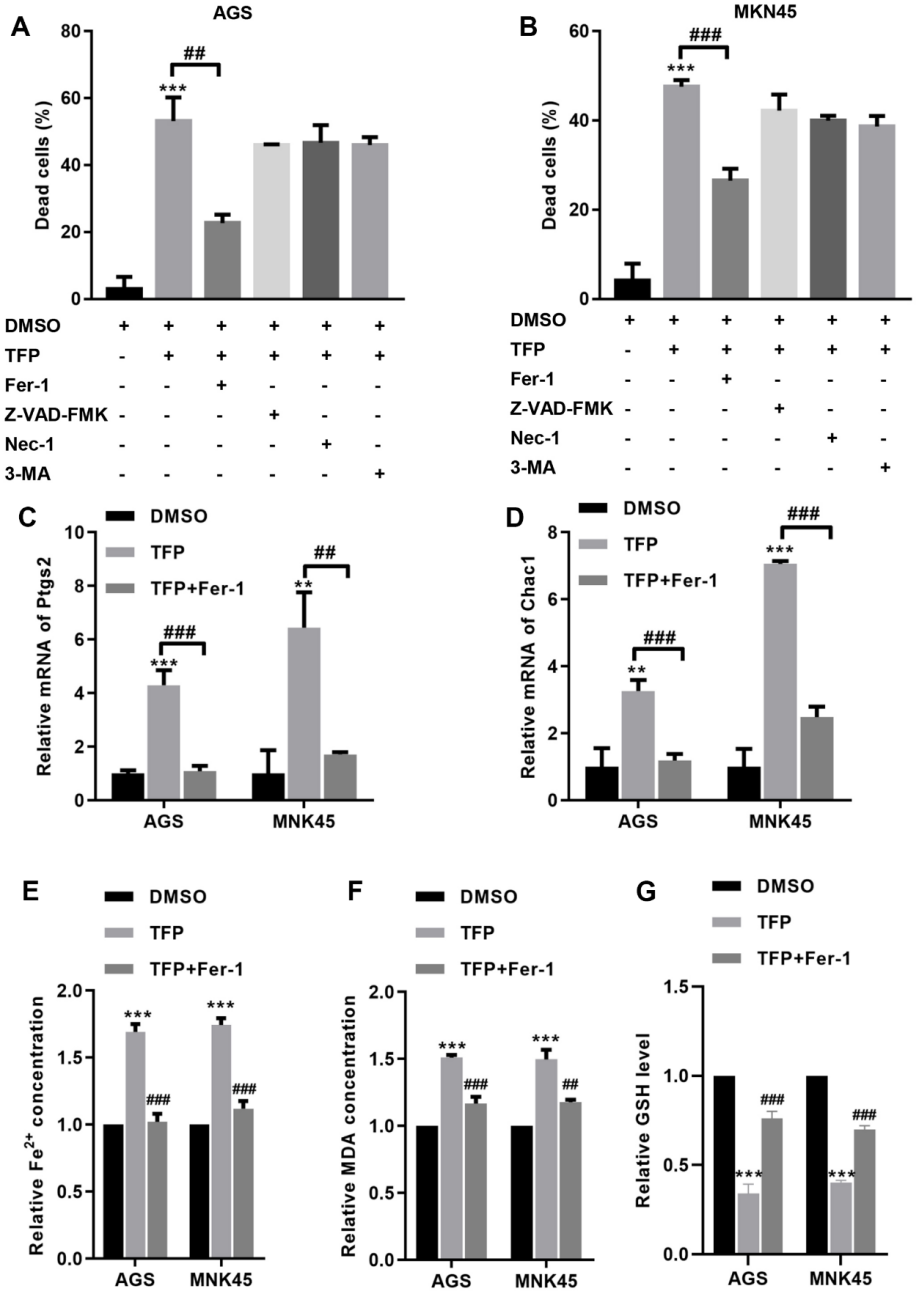
**TFP induced ferroptosis in EBV-infected AGS and MKN45 cells.** EBV-infected AGS and MKN45 cells were preincubated with 1 μM Fer-1, 20 μM Z-VAD-FMK, 20 μM Nec-1 and 20 μM 3-MA for 1 h and then treated with TFP for 24 h. CCK-8 assays showed that TFP elevated EBV-infected AGS (**A**) and MKN45 (**B**) cell death, but preincubation with Fer-1 reduced these effects. RT–PCR analysis indicated that the mRNA levels of Ptgs2 and Chac1 were increased after TFP treatment, but Fer-1 preincubation abolished these effects in AGS (**C**) and MNK45 (**D**) cells. TFP enhanced the levels of Fe^2+^ (**E**) and MDA (**F**) in EBV-infected AGS and MKN45 cells, but Fer-1 preincubation abolished these effects. (**G**) TFP-induced reduction of GSH could also be abolished by Fer-1 in both EBV-infected AGS and MNK45 cells. **p<0.01, ***p<0.001 vs. con; ##p<0.01, ###p<0.001 vs. TFP.

To explore the specific mechanism of TFP-induced cell death, we preincubated EBV-infected AGS and MNK45 cells with different reagents, including Fer-1 (a ferroptosis inhibitor), Z-VAD-FMK (an apoptosis inhibitor), necrostatin-1 (Nec-1, a necrosis inhibitor), and 3-methyladenine (3-MA, an autophagy inhibitor). The CCK-8 assay indicated that TFP elevated EBV- infected AGS and MKN45 cell death by approximately 50% and 47%, but preincubation with Fer-1 reduced TFP-induced cell death to approximately 23% and 26% in EBV-infected AGS and MKN45 cells (Figure 4A, 4B). However, Z-VAD-FMK, Nec-1 and 3-MA did not significantly change TFP-induced cell death in either EBV-infected AGS or MKN45 cells (Figure 4A, 4B). We also tested the effects of TFP on the mRNA levels of Ptgs2 and Chac1, two important ferroptosis markers, in both EBV-infected AGS and MNK45 cells. RT–PCR analysis indicated that the mRNA levels of PTGS2 and Chac1 were increased after TFP treatment, but Fer-1 preincubation abolished these effects in both EBV-infected AGS and MNK45 cells (Figure 4C, 4D). Compared to the control group, the levels of Fe^2+^ and MDA were enhanced in the TFP-treated group, while preincubation with Fer-1 partially reversed these effects in both EBV-infected AGS and MNK45 cells (Figure 4E, 4F). TFP-induced reduction of GSH could also be abolished by Fer-1 in both EBV-infected AGS and MNK45 cells (Figure 4G). These findings suggested that ferroptosis was a major contributor to TFP-induced cell death.

### TFP suppressed NRF2/HO-1 activation in both EBV-infected AGS and MKN45 cells

**Figure 5:**
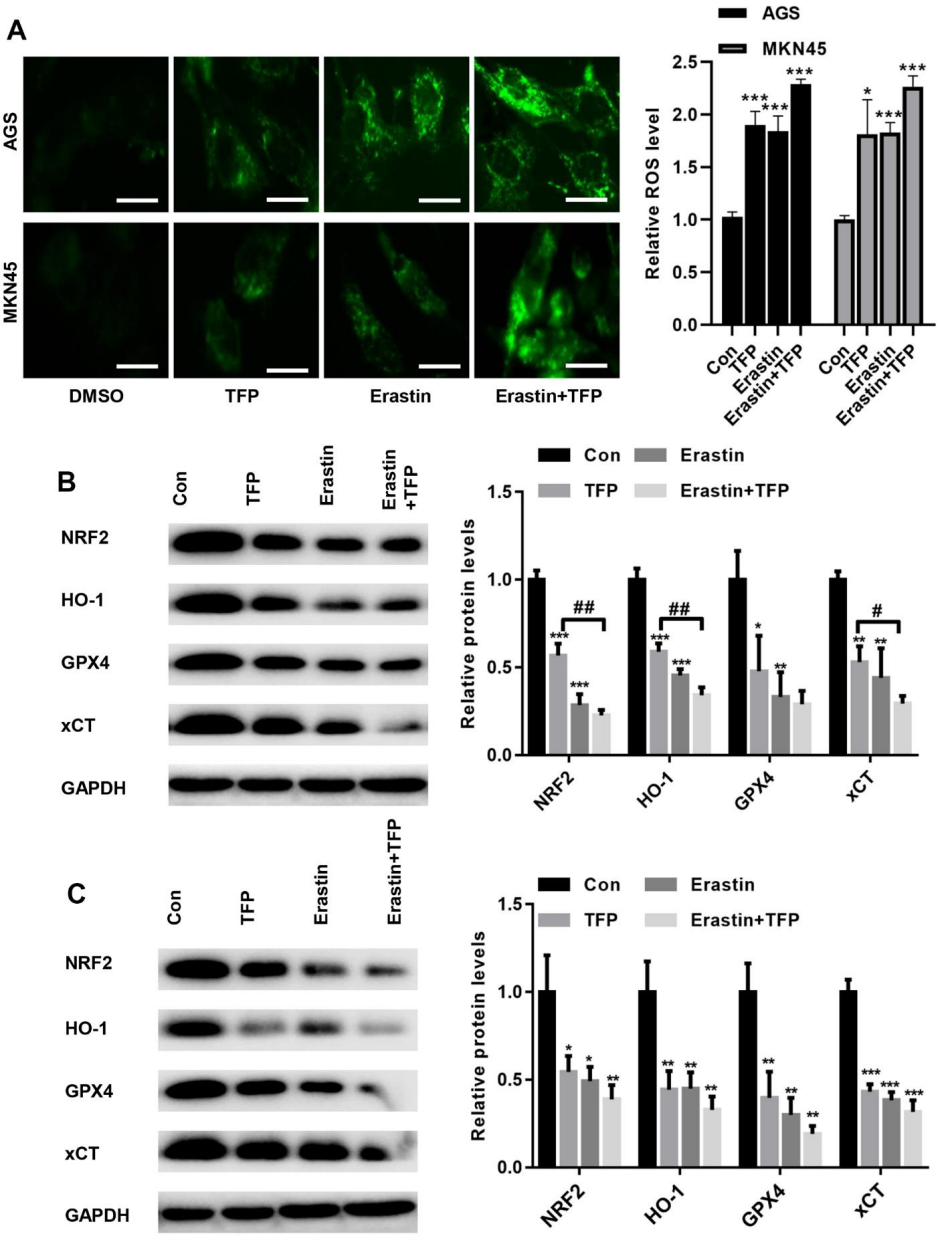
**TFP suppressed NRF2/HO-1 activation in both EBV-infected AGS and MKN45 cells.** EBV-infected AGS and MKN45 cells were preincubated with 40 μg/mL or 20 μg/mL TFP or 10 μM erastin for 24 h. (**A**) DCFH-DA staining showed that TFP elevated ROS production in EBV-infected AGS and MKN45 cells (bar=30 μm). Western blot assays showed that TFP significantly suppressed the expression of NRF2, HO-1, GPX4 and xCT in both AGS (**B**) and MKN45 (**C**) cells. *p<0.05, **p<0.01, ***p<0.001 vs. DMSO; #p<0.05, ##p<0.01 vs. erastin.

To determine the underlying mechanism by which TFP induces ferroptosis, we tested the effects of TFP on ROS production in EBV-infected AGS and MKN45 cells. DCFH-DA staining showed that TFP treatment promoted ROS accumulation in EBV-infected AGS and MKN45 cells (Figure 5A). Consistently, erastin also induced ROS production in both EBV-infected AGS and MKN45 cells (Figure 5A). Moreover, cotreatment with TFP and erastin demonstrated a synergistic effect in EBV-infected AGS and MKN45 cells (Figure 5A). Studies have indicated that NRF2/HO-1 signaling plays a key role in ferroptosis in gastric cancer [24, 25]. Western blot assays showed that TFP significantly suppressed the expression of NRF2, HO-1, GPX4 and xCT in both EBV-infected AGS and MKN45 cells (Figure 5B, 5C). Consistently, the combination of TFP and erastin also decreased the expression of HO-1, GPX4 and xCT in both EBV-infected AGS and MKN45 cells (Figure 5B, 5C).

### TFP-induced ferroptosis was mediated via NRF2 in EBV-infected AGS and MKN45 cells

**Figure 6:**
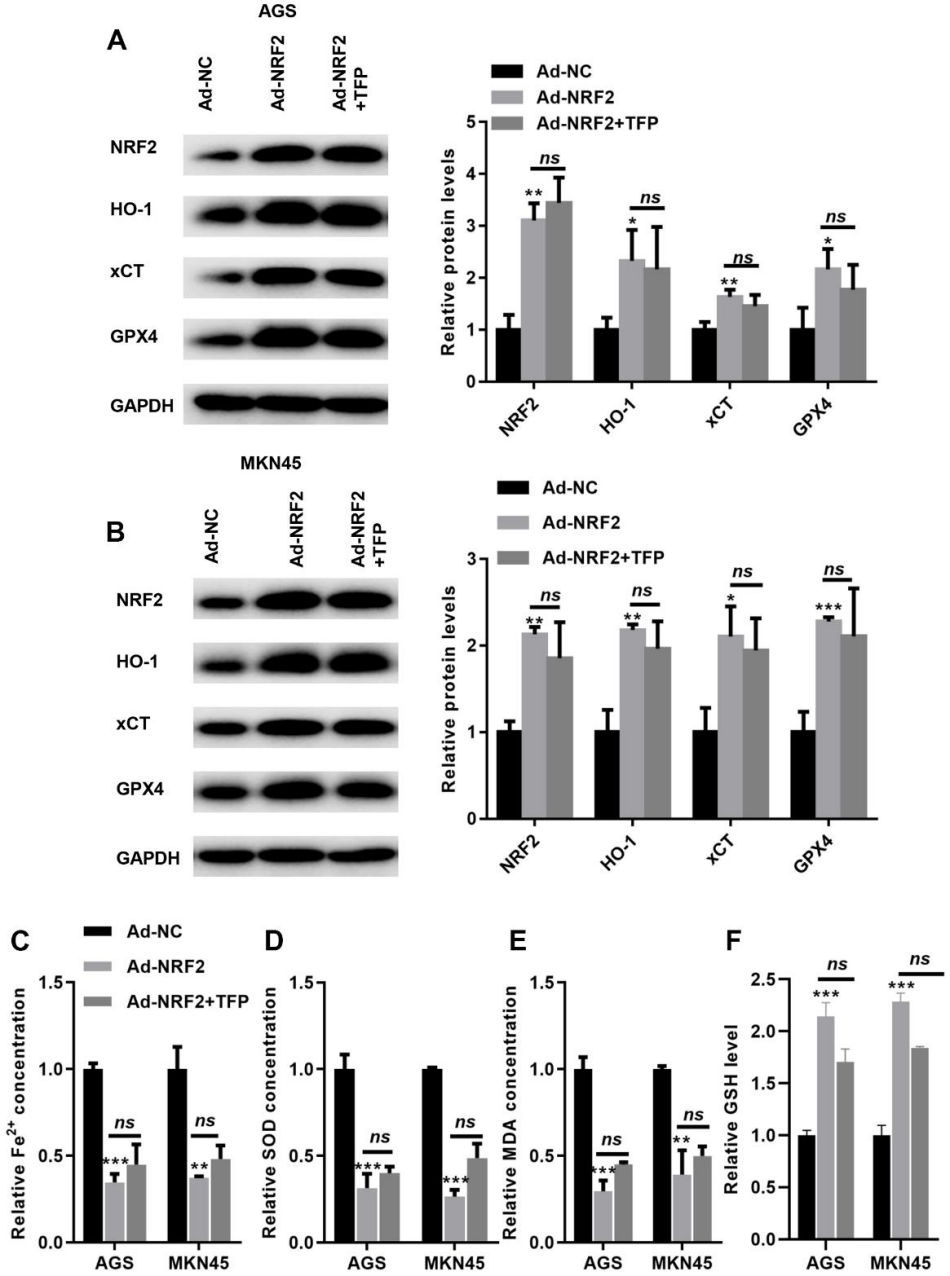
**TFP-induced ferroptosis was mediated via NRF2 in gastric cancer cells.** EBV-infected AGS and MKN45 cells were preincubated with TFP for 2 h. After that, Ad-NRF2 and Ad-NC were transfected into EBV-infected AGS and MKN45 cells for 24 h. Transfection with ad-NRF2 abolished TFP-induced suppression of HO-1, GPX4 and xCT in AGS (**A**) and MKN45 (**B**) cells. Overexpression of NRF2 reduced intracellular Fe^2+^ (**C**), SOD (**D**) and MDA (**E**) levels, even in the presence of TFP, in EBV-infected AGS and MKN45 cells. (**F**) Elevated NRF2 expression increased the level of GSH, and TFP pretreatment could not affect such effects in EBV-transfected GC cells. *p<0.05, **p<0.01, ***p<0.001 vs. con.

We then overexpressed NRF2 in EBV-infected AGS and MKN45 cells. As shown in Figure 6A, 6B, transfection with ad-NRF2 significantly elevated the expression of NRF2 compared with that of ad-NC in EBV-infected AGS and MKN45 cells. Furthermore, upregulation of NRF2 elevated the expression of HO-1, GPX4 and xCT in EBV-infected AGS and MKN45 cells (Figure 6A, 6B). Meanwhile, the suppression of GPX4 and xCT by TFP was obviously rescued by overexpressing NRF2 in gastric cancer cells (Figure 6A, 6B). Furthermore, overexpression of NRF2 reduced intracellular Fe^2+^, SOD and MDA levels, even in the presence of TFP (Figure 6C, 6D, and 6E). In contrast, elevated NRF2 expression increased the level of GSH, and TFP pretreatment could not affect such effects in EBV-transfected GC cells (Figure 6F). These data indicated that TFP-induced ferroptosis was mediated via NRF2 in EBV-transfected GC cells.

## DISCUSSION

EBV infection is closely related to the occurrence and development of some gastric cancers [26]. At present, there is no specific treatment for EBVaGC. Surgical resection or laparoscopic resection is still the main treatment of choice, and the procedure is the same as that for general gastric cancer, and there is no specific sensitive drug for postoperative chemotherapy [26, 27]. Compared to the current better measures and protocols for the prevention and treatment of *Helicobacter pylori* (HP) infection, strengthening the detection and treatment of EBVaGC will bring greater benefits to some patients with gastric cancer and even other common malignancies [26, 27].

Ferroptosis is a form of cell death mediated by iron-dependent oxidative stress that can be induced by erastin and RSL3 [28]. Here, we first explored whether the two cell lines, EBV-infected AGS and MKN45, were sensitive to ferroptosis. Our data showed that erastin treatment significantly reduced cell viability in EBV-infected AGS and MKN45 cells, but inhibition of ferroptosis via Fer-1 significantly augmented cell viability. Furthermore, erastin also decreased the levels of MDA and Fe^2+^ in EBV-infected AGS and MKN45 cells. These observations indicated that EBV-infected AGS and MKN45 cells were sensitive to ferroptotic cell death.

*Tremella fuciformis* is very popular in China due to its nutritive and tonic actions for treating exhaustion [29]. Its nonstarch polysaccharide component, TFP, is characterized by various pharmacological activities, including anticancer, hypoglycemic, anti-inflammatory, and antioxidant functions [30, 31]. However, whether TFP suppresses gastric cancer has never been explored. In the present study, we demonstrated an antitumor role of TFP in gastric cancer cells. The CCK-8 assay showed that TFP decreased EBV-infected AGS and MKN45 cell viability in a dose- and time-dependent manner. Meanwhile, TFP significantly induced EBV-infected AGS and MKN45 cell death. The migratory capacity was also reduced when EBV-infected AGS and MKN45 cells were treated with TFP. To our knowledge, this is the first study to demonstrate the anticancer role of TFP in gastric cancer cells.

We further explored the underlying mechanism by which TFP induced EBV-infected gastric cancer cell death. In contrast to other inhibitors, Fer-1, a ferroptosis inhibitor, significantly reversed TFP-induced cell death. We propose that ferroptosis is a major contributor to TFP-induced gastric cancer cell death. Ferroptosis is recognized as a nonapoptotic regulated form of cell death and is characterized by iron-dependent lipid peroxidation [32]. Erastin is a classical ferroptosis inducer that mainly suppresses cystine uptake and depletes GSH by inhibiting xCT [33]. GSH is a major antioxidant that regulates biologic redox equilibrium and defends against oxidative injury, and it is also a necessary substrate for GPX4 [33, 34]. Once GPX4 is depleted, redox homeostasis may be disturbed, and lipid hydroperoxides are elevated, thereby triggering ferroptosis [35]. In the current study, we validated that treatment with TFP significantly increased the mRNA levels of PTGS2 and Chac1 in EBV-infected AGS and MKN45 cells. Meanwhile, the MDA, Fe^2+^ and ROS levels were enhanced in EBV-infected AGS and MKN45 cells treated with TFP. In comparison, Fer-1, a lipid peroxidation inhibitor, significantly reversed TFP-induced upregulation of MDA, Fe^2+^ and ROS levels in gastric cancer cells. These findings suggested that TFP indeed induced ferroptosis in gastric cancer cells.

NRF2/HO-1 signaling plays a key role in the endogenous antioxidative stress pathway [19]. Under normal conditions, NRF2 interacts with Keap1 and leads to its degradation [19]. Once oxidative stress is induced, NRF2 dissociates from Keap1 and translocates into the nucleus, thereby activating downstream genes, including HO-1 [36]. Accumulating studies have indicated that HO-1 is a key regulator of ferroptosis, and many drugs have been shown to suppress ferroptosis by activating NRF2/HO-1 signaling [36, 37]. For instance, gastrodin protects HT-22 cells from glutamate-induced ferroptosis by activating NRF2/HO-1 signaling [36]. Moreover, melatonin has also been shown to suppress ferroptosis and enhance the osteogenic capacity of osteoblasts by activating Nrf2/HO-1 signaling both *in vivo* and *in vitro* [37]. In this study, the results indicated that TFP suppressed the expression of NRF2, HO-1, GPX4 and xCT in both AGS and MKN47 cells. More importantly, overexpression of NRF2 could obviously rescue TFP-induced downregulation of GPX4 and xCT in gastric cancer cells. An *in vivo* assay also demonstrated that TFP treatment reduced *in vivo* tumor weight and volume. Meanwhile, the MDA and Fe^2+^ levels were decreased by TFP treatment. Western blot assays also showed that the protein levels of NRF2, HO-1, GPX4 and xCT were suppressed by TFP compared with those of controls. These findings suggested that TFP-induced ferroptosis is mainly mediated by inactivating NRF2/HO-1 signaling.

However, there are limitations in the present study. First, we did not do animal experiments to explore whether TFP inhibits tumor growth in mice *in vivo*. Second, whether other modes of death, such as apoptosis, necrosis and autophagy, are involved in TFP-induced EBV-GC cell death deserves further study. Third, TFP is an important component of *Tremella fuciformis*, accounting for approximately 60-70% of the dry weight of psyllium [38]. Our current results confirm that TFP can inhibit the malignant phenotype of EBVaGC *in vitro*, but the use of TFP alone may not be effective in preventing EBVaGC, mainly because EBVaGC is the result of a complex, multi-gene co-regulation. Moreover, there are no clinical studies supporting the therapeutic efficacy of TFP in gastrointestinal diseases. Therefore, more studies are needed to confirm the anti-EBVaGC effect of TFP in the future.

**Figure 7:**
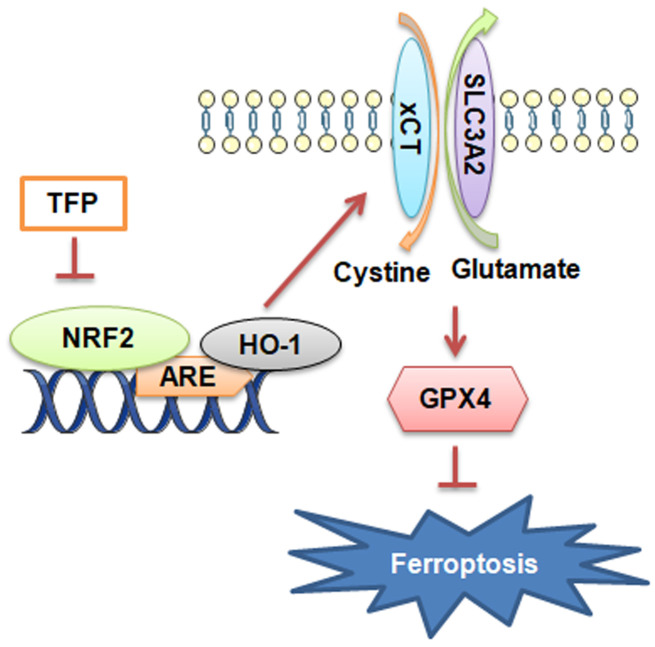
Diagram of the molecular mechanism by which TFP inhibits EBVaGC.

Collectively, the present study showed novel data that TFP was a strong inducer of ferroptosis in EBV-infected gastric cancer cells and that such effects were achieved by inhibiting NRF2/HO-1 signaling (Figure 7). EBVaGC is a gastric cancer subtype with unique pathologic features based on its high expression of immune checkpoint proteins and high lymphocytic infiltration characteristics. In this study, we found for the first time that TFP is expected to be a new therapeutic strategy for EBVaGC. However, clinical studies of TFP for the treatment of EBVaGC are not yet available, and its specific efficacy needs to be further verified.

## MATERIALS AND METHODS

### Cell culture

MKN45 and AGS cells were purchased from Procell (Wuhan City, China, https://www.procell.com.cn/). Cell authentication was performed using STR profiling. MKN45 cells were cultured in RPMI-1640 medium, and AGS cells were cultured in Ham’s F-12 medium. AGS and MKN45 cells were infected with EBV-Akata recombinant virus carrying the neomycin-resistance gene and selected by G418 (Roche Diagnostics, Basel, Switzerland) at 200 μg/mL, as previously reported [39]. All culture medium was supplemented with 0.1 mg/mL streptomycin (GE Healthcare, Logan, UT, USA), 100 units/mL penicillin (GE Healthcare, Logan, UT, USA), and 10% fetal bovine serum (FBS, Gibco, Waltham, MA, USA) at 37° C in a humidified atmosphere with 5% CO_2_.

### Construction of adenovirus vectors

Adenovirus vectors overexpressing NRF2 (ad-NRF2) or blank vector were constructed by WZ Biosciences Inc. (http://www.wzbio.com.cn/, Jinan, Shandong, China).

### Cell counting kit-8 (CCK-8) assay

Cell viability was determined using the CCK-8 assay in EBV-infected AGS and MKN45 cells. In brief, EBV-infected AGS and MKN45 cells were seeded in 96-well plates at a density of 3,000 cells/well for 24 h. Then, the cells were incubated with TFP (ST8770, Solarbio, Beijing, China) at a density of 0, 5, 10, 25, 50, 100, and 200 μg/mL and 0, 1, 2, 4, 6, 8, 16, 32 and 64 μg/mL in EBV-infected AGS and MKN45 cells for 24 h, respectively. Then, 10 μL of CCK-8 solution was added to each well for 4 h, and cell viability was determined at OD_450 nm_ by applying an ELISA browser (Bio-Tek EL 800, Winooski, VT, USA).

### Quantification of malondialdehyde (MDA), glutathione (GSH) and Fe^2+^

EBV-transfected AGS and MKN45 cells were seeded in six-well plates at a density of 5×10^5^ cells/well overnight. The cells were preincubated with 1 μM ferrostatin-1 (Fer-1, HY-100579, MCE, USA) for 1 h. After that, the cells were treated with 20 and 40 μg/mL TFP or 10 μM erastin (HY-15763, MCE) for 24 h. The cells were collected, and intracellular MDA, GSH and Fe^2+^ levels were quantified using a Lipid Peroxidation MDA Assay Kit (S0131, Beyotime Biotechnology, Beijing, China), GSH assay kit (BC1175, Solarbio, Beijing, China) and iron assay kit (ab83366, Abcam, Cambridge, UK), according to the instructions.

### Flow cytometry assay

Dead cells were quantified using an Annexin V-FITC/7-AAD assay kit (P-CA-202, Procell, Wuhan, China). EBV-transfected AGS and MKN45 cells were seeded in six-well plates at a density of 5×10^5^ cells/well overnight. The cells were treated with 20 and 40 μg/mL TFP for 24 h. Then, the cells were collected and resuspended in 500 μL binding buffer. Subsequently, 5 μL Annexin V-APC and 5 μL 7-AAD were added and mixed at room temperature for 10 min. Dead cells were quantified using an FC500 flow cytometry instrument equipped with CXP software (Beckman Coulter, Bethesda, MA, USA). According to the instructions, Q2 represented necrosis and late apoptotic cells, while Q3 represented early apoptotic cells. Hence, only cells in the Q2 region were calculated in the present study.

### Transwell assay

Transwell assays were used to determine the migration capacity of EBV-transfected AGS and MKN45 cells based on Transwell plates (Corning, Inc., Corning, NY, USA) according to the manufacturer’s instructions. Briefly, EBV-infected AGS and MKN45 cells were seeded in upper chambers at a density of 10^5^ cells/well overnight. The cells were treated with 20 and 40 μg/mL TFP for 24 h. In the lower chamber, fresh medium containing 20% FBS was added. After incubation for 24 h at 37° C, the migratory cells were stained with 0.1% crystal violet (Solarbio, Beijing, China) at room temperature for 20 min. Five visual fields were randomly selected to observe the number of cells under a microscope (Olympus Corporation, Tokyo, Japan).

### 2,7-Dichlorodihydrofluorescein diacetate (DCFH-DA) staining

EBV-infected AGS and MKN45 cells were seeded in six-well plates at a density of 5×10^5^ cells/well overnight. The cells were treated with 20 and 40 μg/mL TFP and/or 10 μM erastin for 24 h. After that, the cells were incubated with 1 mL of DCFH-DA (1:1000, D6470, Solarbio, Beijing, China) at 37° C for 20 min. Subsequently, the cells were washed with fresh culture medium without FBS three times (5 min/time) at room temperature. Intracellular ROS were observed under a fluorescence microscope (Olympus Corporation, Tokyo, Japan).

### Western blot

Total proteins were isolated from EBV-infected AGS or MKN45 cells using RIPA buffer (Solarbio, Beijing, China). The protein concentration was determined using a BCA assay kit (Pierce; Thermo Fisher Scientific, Inc., Waltham, MA, USA). The protein was isolated using 12% SDS–PAGE and transferred onto PVDF membranes (Millipore, Burlington, MA, USA). The membranes were blocked with 5% milk (Pierce; Thermo Fisher Scientific, Inc.) and washed with PBST three times (5 min/time). The membranes were incubated with the following primary antibodies at 4° C overnight: NRF2 (12721, 1:1000, Cell Signaling Technology, Inc., USA), HO-1 (26416, 1:1000, Cell Signaling Technology, Inc.), GPX4 (52455, 1:1000, Cell Signaling Technology, Inc.), xCT (ab175186, 1:1,000; Abcam, Cambridge, UK), and GAPDH (5174, 1:5,000; Cell Signaling Technology, Inc.). The membranes were then incubated with horseradish peroxidase (HRP)-conjugated goat anti-rabbit IgG (both 1:5,000; cat. no. ZB-2301; Beijing Zhongshan Golden Bridge Biotechnology Co., Beijing, China) for 2 h at room temperature, followed by three washes with TBST. Enhanced chemiluminescence (EMD Millipore, Billerica, MA, USA) was used to determine the protein concentrations according to the manufacturer’s protocol. Signals were detected using an Ultra High Sensitivity ECL Substrate Kit (ab133409; Abcam, Cambridge, UK), and quantitative analysis was performed using UVP 7.0 software (UVP LLC, Upland, CA, USA). Relative protein expression was normalized to GAPDH. All experiments were repeated three times. ImageJ 1.43b software (National Institutes of Health, Bethesda, MD, USA) was used for densitometry analysis.

### RT–qPCR

Total RNA was isolated from EBV-infected AGS or MKN45 cells using TRIzol reagent (Solarbio, Beijing, China). Total RNA (1.0 μg) was reverse-transcribed to first-strand cDNA using Moloney murine leukemia virus reverse transcriptase (NEB, Ipswich, MA, USA) according to the manufacturer’s instructions. The primers were as follows: PTGS2-F: GAGGGATCTGTGGATGCTTCG; PTGS2-R: AAACCCACAGTGCTTGACAC; Chac-1-F: GGAACTTGACCAGATTCCCCC; Chac-1-R: AGAGGATCGAGGCTCTTGGA; GAPDH-F: TTCAACAGCGACACCCACTC; GADPH-R: TGGTGGTCCAGGGGTCTTAC. Quantitative real-time PCR was carried out using the iQ5 Optical System (Bio-Rad Laboratories, Hercules, CA, USA) in combination with SYBR Green (Roche Applied Science, Mannheim, Germany). The procedures were as follows: 95° C for 30 s and 40 cycles at 95° C for 30 s, 60° C for 30 s, and 72° C for 30 s. GAPDH was used as an internal control, and the data presented are the average of three independent experiments. Relative mRNA expression was normalized to GAPDH using the 2^-∆∆Cq^ method [40].

### Statistical analysis

Data are expressed as the mean ± SD, and the statistical analysis was conducted using GraphPad Prism 7. The unpaired Student’s *t* test was used to compare two groups, and one-way ANOVA followed by *post hoc* analysis was used to compare multiple groups. *P* < 0.05 was considered statistically significant.
